# The SNP rs13147758 in the *HHIP* Gene Is Associated With COPD Susceptibility, Serum, and Sputum Protein Levels in Smokers

**DOI:** 10.3389/fgene.2020.00882

**Published:** 2020-09-24

**Authors:** Alejandro Ortega-Martínez, Gloria Pérez-Rubio, Enrique Ambrocio-Ortiz, Karol J. Nava-Quiroz, Rafael de Jesus Hernández-Zenteno, Edgar Abarca-Rojano, Sebastián Rodríguez-Llamazares, Andrea Hernández-Pérez, Leonor García-Gómez, Alejandra Ramírez-Venegas, Ramcés Falfán-Valencia

**Affiliations:** ^1^HLA Laboratory, Instituto Nacional de Enfermedades Respiratorias Ismael Cosío Villegas, Mexico City, Mexico; ^2^Sección de Estudios de Posgrado e Investigación, Escuela Superior de Medicina, Instituto Politécnico Nacional, Mexico City, Mexico; ^3^Tobacco Smoking and COPD Research Department, Instituto Nacional de Enfermedades Respiratorias Ismael Cosío Villegas, Mexico City, Mexico

**Keywords:** COPD, tobacco smoking, SNPs, HHIP protein levels, sputum

## Abstract

**Background:**

Genetic association studies have identified single nucleotide polymorphisms (SNPs) related to chronic obstructive pulmonary disease (COPD) susceptibility. The aim of this study was to identify *HHIP* genetic variants associated with COPD, pulmonary function, and serum and sputum HHIP protein levels in Mexican mestizo smokers.

**Materials and Methods:**

Association analysis was performed by carrying out a case–control study in Mexican mestizo smokers comprised of two groups: tobacco-smoking subjects with COPD (COPD-TS, *n* = 222) and smokers without COPD (SWOC, *n* = 333). We evaluated three SNPs (rs13147758, rs1828591, and rs13118928) in the *HHIP* gene. Allele discrimination was accomplished by qPCR using TaqMan probes, and determination of protein levels in the serum and sputum supernatants (SS) was performed using ELISA.

**Results:**

Statistically significant differences were observed in the rs13147758 GG genotype (adjusted *p* = 0.014, OR = 1.95) and the rs13147758-rs1828591 GA haplotype (*p* = 6.6E-06, OR = 2.65) in the case–control comparison. HHIP protein levels were elevated in SS samples from the COPD-TS group compared to those from the SWOC group (*p* = 0.03). Based on genotype analysis, HHIP protein levels were lower in the serum samples of rs13147758 GG genotype carriers in the COPD-TS group than in the serum samples of rs13147758 GG genotype carriers from the SWOC group (*p* < 0.05), but there were no differences in the sputum samples.

**Conclusion:**

The rs13147758 GG genotype and the rs13147758-rs1828591 GA haplotype are associated with susceptibility to COPD. Furthermore, an association in protein levels was observed between the *HHIP* rs13147758 genotype and COPD in Mexican mestizo smokers.

## Introduction

Tobacco smoking (TS) is considered the leading risk factor for the development of chronic obstructive pulmonary disease (COPD), as approximately 50% of COPD cases worldwide are related to TS (Lamprecht et al., 2011; Bajpai et al., 2019). However, only 15–20% of smokers develop the disease, and previous studies suggest that genetic variants contribute to COPD susceptibility (Nakamura, 2011).

Through genome-wide association studies (GWAS), it is possible to examine millions of genetic variants, mostly single nucleotide polymorphisms (SNPs), and to identify different genomic regions related to pulmonary function traits, emphysema, and several phenotypes of COPD (Hobbs and Hersh, 2014). Pillai et al. (2009) identified genetic variants in the hedgehog interacting protein (*HHIP*) gene that are linked with COPD; however, the relationships did not reach statistical significance at the GWAS level. Subsequently, Wilk et al. identified *HHIP* SNPs associated with decreased forced expiratory volume in the first second (FEV_1_) in a general population in the Framingham Heart Study (Hobbs and Hersh, 2014).

The *HHIP* gene encodes the Hedgehog interacting protein, which belongs to the hedgehog family of genes and plays a role in lung morphogenesis and development (Nakamura, 2011). The *HHIP* gene, which contains 13 exons, has a length of >91 kb, is located on chromosome 4q31.21, and encodes a protein of 700 amino acids (Bak et al., 2001). Recent investigations have identified several SNPs in the non-coding regions of the *HHIP* gene that are associated with COPD susceptibility and other pulmonary function traits, primarily in Caucasian and Asian people; however, mixed populations (defined as mestizos, as a product of two or more ancestral contributions) have not been evaluated. We hypothesized that SNPs in the *HHIP* gene set convey genetic differences related to susceptibility to COPD secondary to TS. We aimed to identify possible associations between three SNPs (rs13147758, rs13118928, and rs1828591) in the *HHIP* gene and COPD susceptibility, pulmonary function traits, and HHIP protein levels in the serum and sputum in Mexican mestizo smokers.

## Materials and Methods

### Case and Control Groups

Five hundred fifty-five Mexican-Mestizo participants were included in this case–control study. These subjects attended COPD and smoking cessation support clinics, both of which were part of the Department of Smoking and COPD Research Department of the Instituto Nacional de Enfermedades Respiratorias Ismael Cosio Villegas (INER), Mexico.

Applying diagnostic criteria according to the Global Initiative for Chronic Obstructive Lung Disease (GOLD) guidelines (Global Initiative for Chronic Obstructive Lung Disease [GOLD], 2013) and considering the symptoms and the deterioration of patient health status, a specialized chest physician team completed the clinical evaluations. The diagnosis was confirmed using lung function tests (by postbronchodilator spirometry), with a ratio of forced expiratory volume in the first second/forced vital capacity (FEV_1_/FVC) < 70% indicating COPD according to the reference values for Mexicans obtained by Perez-Padilla et al. (Pérez-Padilla et al., 2006).

Participants were older than 40 years, current or former smokers, who had quit smoking for less than one year before being enrolled in the study. Patients with a tobacco index (TI) ≥ 10 (≥10 cigarettes per day, ≥10 years of smoking), and patients who had never been exposed to biomass burning smoke (or other fumes or gases associated with COPD development) composed the COPD-TS group.

All included patients were clinically stable, did not require supplementary oxygen at the time of enrollment, did not have a history of previous exacerbations, and did not undergo antibiotic or systemic corticosteroid treatment in the three months prior to enrollment. Consecutive COPD patients from the COPD support clinic were enrolled from 2009 to 2016. GOLD stage I and II patients were grouped as G1, while stage III and IV patients were considered G2.

The control group consisted of smokers without COPD (SWOC, *n* = 333), including current and former smokers who had quit smoking for less than one year, had no clinical evidence of pulmonary disease, possessed normal spirometry parameters (FEV_1_/FVC > 70%), and had no evidence of other respiratory or chronic inflammatory diseases.

All participants completed a questionnaire regarding family and inherited pathologies; participants who reported suffering pulmonary (other than COPD) or chronic inflammatory diseases and those with no Mexican ancestry (no Mexican-by-birth parents or grandparents) were excluded. Those with ancestors who were born in Mexico for at least three previous generations were considered Mexican mestizo. All participants were selected from the HLA Laboratory biobank, which has been previously analyzed for population structure employing an AIMs set. According to this analysis, there is no statistical significance (Fst = 0) among groups (Pérez-Rubio et al., 2016). Participants had no biological relation with any of the subjects in the same or corresponding comparison group and no family history of pulmonary diseases.

### Ethical Approval and Informed Consent

This study was reviewed and accepted by the Institutional Committees for Investigation, Ethics in Research, and Biosecurity of the Instituto Nacional de Enfermedades Respiratorias Ismael Cosío Villegas (INER) (approbation number: B11-19). All participants were informed of the study protocol’s aim after being given a detailed description of the study and invited to participate as volunteers. All subjects signed informed consent forms and were supplied with a privacy statement describing the legal protection of personal data. Both documents were approved by the Institutional Research and Ethics in Research Committees. All analyses were conducted following the relevant guidelines and regulations. STREGA (STrengthening the REporting of Genetic Association) (Little et al., 2009) recommendations were taken into consideration in the design of this genetic association study.

### Blood Sample Processing and DNA Extraction

Sample processing began with a 15-mL sample of whole-blood obtained by venipuncture collected in two EDTA tubes (S-Monovette 4.9 ml K3E, Sarstedt, Nümbrecht, Germany) and another tube for serum obtention (S-Monovette 4.9 ml Z-Gel, Sarstedt, Nümbrecht, Germany). Subsequently, the samples were centrifuged for 5 min at 4,500 rpm to separate the peripheral blood mononuclear cells (PBMCs) and the serum, and the serum was stored in cryopreservation tubes at −80°C until use.

### Sputum Induction and Sample Preparation

Based on genotyping analysis, we selected a subsample of participants for more in-depth characterization. To obtain sputum, we followed a previously published protocol (Ambrocio-Ortiz et al., 2020). Briefly, participants were nebulized with a sterile 7% saline solution. Treatment lasted for five minutes and was followed by a rest period of five minutes. The treatment and rest cycle was repeated three times. Samples were mechanically disaggregated in equal volumes of 1X PBS buffer (Invitrogen; Carlsbad, CA, United States) to eliminate excess mucus and then centrifuged at 4,500 rpm for 10 min. Then, the saliva was removed, 10 mL of sterile 0.9% saline solution was added, and the sample was centrifuged again at 4,500 rpm for 10 min. Finally, the supernatant was separated into 1.8-mL aliquots. These aliquots were concentrated using a Speedvac concentrator (Thermo Fisher Scientific, Asheville NC, United States) at 14,000 rpm for 12 h, resuspended in 1 mL of 1X PBS, and stored at −80°C until use.

To obtain cells, the remaining sputum was disaggregated in 2 mL of DL-dithiothreitol (DTT, Sputolysin, cat. 560000; Calbiochem Corp, San Diego, CA, United States) by vortexing for 1 min and then mechanically disaggregated in a syringe (10 mL, 20Gx32) 20–25 times. The sample was subsequently centrifuged at 4,500 rpm for 10 min, and the DTT was discarded. The samples were washed three times with 10 mL 1× PBS, and after each wash, samples were centrifuged at 4,500 rpm for 10 min. Between each wash, the samples were filtered through a 70-μm cell strainer (ref. 352350, Corning Inc. Durham, NC, United States), and the supernatant, including mucus traces, was discarded at the end of the three washes. The resulting pellet was resuspended in 1.8 mL of 1× PBS and stored at −80°C until use.

### SNP Selection

**FIGURE 1:**
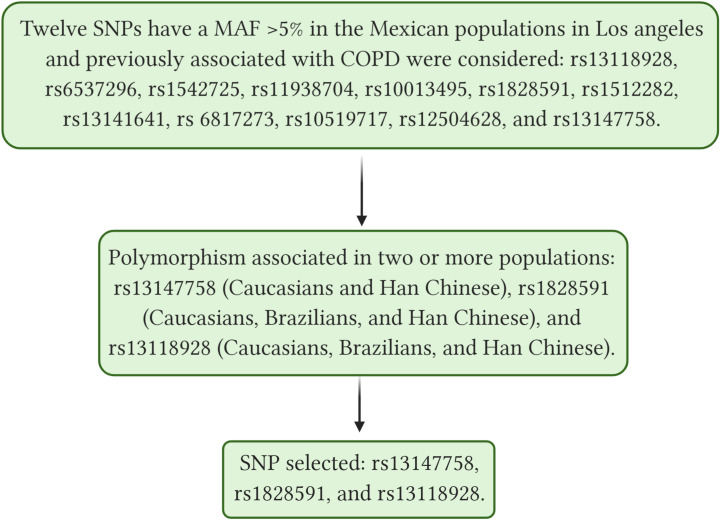
Shows selection process of the SNPs, SNP, single nucleotide polymorphism; COPD, Chronic Obstructive Pulmonary Disease; MAF, minor frequency allele. rs13118928, 2020b; rs6537296, 2020; rs1542725, 2020; rs11938704, 2020; rs1512282, 2020; rs13141641, 2020; rs10519717, 2020; rs12504628, 2020; rs13147758, 2020b; rs1828591, 2020b. Created with BioRender.com.

Single nucleotide polymorphisms were selected based on a bibliographic search of the National Center for Biotechnology Information (NCBI) database and by identifying polymorphisms previously associated with COPD in different GWAS with positive replication in at least two other populations. In addition, we considered SNPs with a minor allele frequency (MAF) higher than 5% in the Mexican population in Los Angeles, according to the 1000 genomes project (Zerbino et al., 2017). Figure 1 shows the selection process of the SNPs.

### SNP Genotyping

Allele discrimination of SNP variants was performed using commercial TaqMan probes (Applied Biosystems, CA, United States) at a concentration of 20X. We selected three SNPs: rs13118928 (commercial probe id: C__11375931_20), rs1828591 (C__11482211_10), and rs13147758 (C___2965080_10). These SNPs were identified in intronic (non-coding) regions. Supplementary Table S1 summarizes the principal characteristics of the assessed SNPs.

Genotyping was performed by real-time PCR (qPCR) in a StepOne Real-Time PCR System (Applied Biosystems/Thermo Fisher Scientific Inc., Singapore) and genotype assignment with sequence detection software (SDS) version 2.3 (Applied Biosystems, CA, United States).

### Serum and Sputum HHIP Protein Level Determination

Enzyme-linked immunosorbent assay (ELISA) was utilized for the determination of protein levels in serum (*n* = 80) and sputum supernatant (SS) samples (*n* = 40) by employing a commercial kit (cat. E-EL-H0888. Elabscience, Houston, TX, United States) according to the manufacturer’s specifications. This ELISA kit used the Sandwich-ELISA principle. The micro ELISA plate provided in this kit was precoated with an antibody specific for human HHIP. The detection range was 0.31–20 ng/mL, and the sensitivity was 0.19 ng/mL. Assays were performed in duplicate on the same plate. Serum and SS were centrifuged at 1,000 × *g* for 15 min at 6°C before 200 μL of each sample was tested.

### Statistical Assessment

To analyze demographic and clinical characteristics, pulmonary function data, protein levels, and correlations, we used SPSS v.24.0 (IBM, New York, United States) statistical software, and the median, minimum, and maximum values were determined for each continuous quantitative variable.

Before performing genotype comparisons, the Hardy–Weinberg equilibrium (HWE) was calculated using PLINK software v1.9 (Purcell et al., 2007), and De Finetti diagrams were constructed using Finetti v.3.0.8 software. Analysis of the genetic association between groups was performed by comparing allele, genotype, and haplotype frequencies with Pearson’s Chi-squared and Fisher’s exact tests using Epi Info v. 7.1.4.0 (CDC, 2010), Epidat statistical software version 3.1 (Xunta de Galicia Organización Panamericana de la Salud, 2006), Haploview v4.2 (Barrett et al., 2005), and R version 3.6.2 (2019-12-12).

The results were considered statistically significant when the *p*-value was <0.05. Similarly, the odds ratio (OR) with a 95% confidence interval (CI) was estimated to determine the strength of the association.

Logistic regression was performed to adjust for potential confounder variables using Plink v. 1.09 (Purcell et al., 2007) One degree of freedom was created, including age, sex, BMI, and tobacco index as covariables for all results with statistical significance.

## Results

The clinical and demographic characteristics of the participants are shown in Table 1. The median age of patients in both groups was ∼60 years, and smokers with COPD were older than those who did not have COPD (66 vs. 56 years old, *p* < 0.001). The opposite trend was observed for body mass index (BMI), which was ∼25 in the COPD-TS group and ∼27 in the SWOC group (*p* < 0.001). Regarding TS variables, the number of cigarettes per day, years of smoking, and tobacco index were higher in the COPD group than the control group (*p* < 0.05). In contrast, for smoking onset, although smokers with COPD started smoking at an earlier age than smokers without the disease, the difference was not statistically significant (*p* = 0.208). The observed differences in lung function values were expected since postbronchodilator spirometry, and its associated parameters are the diagnostic criteria for differentiating a case from a control.

**TABLE 1:** Demographic, exposition, and lung function characteristics of smokers with and without COPD.

**Variables**	**COPD-TS (*n* = 222)**	**SWOC (*n* = 333)**	***p***
Age (years)	66 (45–88)	56 (40–79)	<0.001
Males (%)	168 (75.7)	146 (43.8)	<0.001
Females (%)	54 (24.3)	187 (56.2)	<0.001
BMI	24.84 (15-57–40.59)	27.40 (15.62–44.44)	<0.001
Years of smoking	40 (3–68)	35 (8–60)	<0.001
Cigarettes/day	20 (20–80)	14 (4–100)	<0.001
Tobacco index	39.42 (10.5–180)	25 (10.20–175)	<0.001
Smoking onset (age)	17 (7–65)	17 (10–50)	0.208
Lung function			
FEV_1_	57 (12–123)	99 (55–134)	<0.001
FVC	82.5 (24–155)	95 (49–144)	<0.001
FEV_1_/FVC	53.6 (19–68.3)	81 (70–112)	<0.001
GOLD			
GOLD I (%)	36	NA	
GOLD II (%)	102	NA	
GOLD III (%)	63	NA	
GOLD IV (%)	21	NA	

*COPD-TS, tobacco-smoking patients with COPD; SWOC, smokers without COPD; BMI, body mass index; FEV_1_, forced expiratory volume in the first second; FVC, forced vital capacity; statistical significance: *p*-value < 0.05; NA: not applicable. The median and min-max values are shown.*

### Genetic Susceptibility Testing

One SNP (rs13118928) was not in HWE (*p* < 0.05), and for this reason, no additional analyses were conducted for this polymorphism. The remaining variants were both in HWE (*p* > 0.1). A comparison between the COPD-TS and SWOC groups indicated that the more common allele was A (adenine) for all polymorphisms, while the minor allele was G (guanine). These findings are similar to previous reports in population databases (rs10013495, 2020). For rs1828591, the difference (in percentage) in the MAF between the groups was not higher than 3%. There were no statistically significant associations for this genetic variant. In contrast, the rs13147758 genotype GG was associated with an increased risk for COPD (*p* = 0.0021). Additionally, a logistic regression analysis adjusted by potential confounding variables (age, sex, IBM, and tobacco index) was performed. Once again, the rs13147758 GG genotype was associated with risk (adjusted *p* = 0.014, OR = 1.95; 95% CI 1.27–2.99). As reported in the unadjusted model, no other SNPs were statistically significant. Table 2 shows these data.

**TABLE 2:** Genotype frequencies among the study groups.

**SNP**	**Genotype**	**COPD-S**		**SWOC**		***p*-value**	**OR**	**95% CI**	*p-value
		***n* = 222**	**%**	***n* = 333**	**%**				
rs13147758	AA	71	31.99	141	42.34	0.016	0.64	0.44–0.91	
	AG	94	42.34	142	42.64	1.0	0.98	0.70–1.39	
	**GG**	**57**	**25.67**	**50**	**15.02**	**0.0021**	**1.95**	**1.27**–**2.99**	**0.014**
rs1828591	AA	87	39.19	143	42.94	0.42	0.85	0.60–1.21	
	AG	100	45.04	141	42.34	0.54	1.11	0.79–1.57	
	GG	35	15.77	49	14.72	0.80	1.08	0.67–1.73	0.68

COPD-TS, tobacco-smoking patients with COPD; SWOC, smokers without COPD; SNP, single nucleotide polymorphism; OR, odds ratio; 95% CI, 95% confidence interval; *p-value adjusted by age, sex, IBM and tobacco index. Associations are shown in the full genotype model.

### Genetic Association Models

According to the data on allele and genotype frequencies, an analysis by genetic models was performed for the two polymorphisms. We found that rs13147758 retained an association with risk in the codominant (OR = 2.26) and recessive (OR = 1.95) models, even after adjusting for potential confounders of age, sex, BMI, and tobacco index (adjusted *p* = 0.012, OR = 2.26, and 95% IC = 1.41–3.64 and *p* = 0.028, OR = 1.95, and 95%IC = 1.27–2.99) for the codominant and recessive models, respectively. Table 3 shows these data in detail. Genetic association models for the remaining SNPs are shown in Supplementary Table S2.

**TABLE 3:** Analysis by genetic models of rs13147758 in smokers with and without COPD.

**Model/Genotype**	**COPD-TS**		**SWOC**		***p*-value**	**OR**	**95% CI**
	***n* = 222**	**%**	***n* = 333**	**%**			
**Codominant**							
AA	71	31.99	141	42.34		1 (ref.)	
AG	94	42.34	142	42.64		1.31	0.89–1.94
GG	57	25.67	50	15.02	0.012*	2.26	1.41–3.64
**Recessive**							
GG	57	25.68	50	15.02	0.028*	1.95	1.27–2.99
AA + AG	165	74.32	283	84.98		0.51	0.33–0.78

COPD-TS, tobacco-smoking patients with COPD; SWOC, smokers without COPD; SNP, single nucleotide polymorphism; OR, odds ratio; 95% CI, 95% confidence interval; statistical significance: *p*-value < 0.05; *p-value adjusted by age, sex, and tobacco index in the recessive model.

### Haplotypes

**FIGURE 2:**
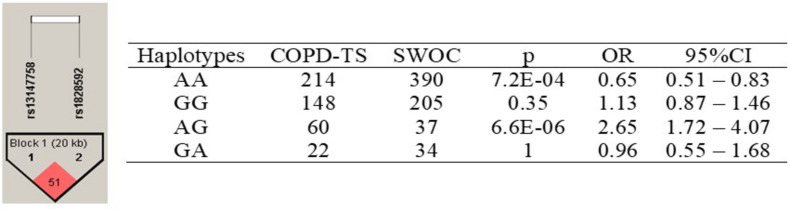
Haplotypes in the *HHIP* gene among COPD-TS and SWOC groups. COPD-TS, tobacco smoking patients; SWOC, Smokers without COPD; OR, Odds ratio; 95%CI, 95% confidence Interval. Statistically *p*-value <0.05; Showing *r*^2^ values among SNPs

The identified haplotypes are shown in Figure 2. Among rs13147758 and rs1828591, there was linkage disequilibrium (*r*^2^ = 0.51).

Four haplotypes were identified. Only the AA haplotype was associated with a decreased risk of COPD (*p* = 7.2E-04, OR = 0.65, 95% CI = 0.51–0.83). All other haplotypes, for example, the AG haplotype (*p* = 6.6E-06, OR = 2.65, 95% CI = 1.72–4.07), were associated with disease susceptibility.

### COPD Severity and Genetic Analysis

Patients were next grouped according to the GOLD stage. Subjects in GOLD stages I and II (*n* = 138) were clustered as G1, while those in GOLD stages III and IV (*n* = 84) were designated G2. A genetic analysis looking for alleles and genotypes associated with more severe cases (G2 vs. G1) was conducted. In the comparison of genotype and allele frequencies between G1 and G2, no statistically significant differences were observed. These data can be found in Supplementary Table S3.

### Serum HHIP Protein Levels in Smokers With and Without COPD

From the initial groups, 80 smokers – 40 with COPD (COPD-TS) and 40 without COPD (SWOC) – were selected for quantification of HHIP protein serum levels.

The median age of patients in both subgroups was 67 years (*p* = 0.29). The COPD-TS group primarily consisted of men (90%), while in the SWOC group, only 50% of patients were men. In the lung function test, a notable difference in the three primary parameters for diagnosis and classification was observed between patients and controls. Patients in the most severe GOLD stages (III, *n* = 25, and IV *n* = 15) were included to ensure protein detection. Supplementary Table S4 shows the clinical and demographic variables of subjects included in this analysis.

The serum protein levels of smokers with COPD were slightly higher than those of smokers without the disease; however, these differences were not statistically significant (*p* = 0.582, Supplementary Figure S1).

### Comparison of HHIP Levels in Patients and Controls According to HHIP Genotypes

**FIGURE 3:**
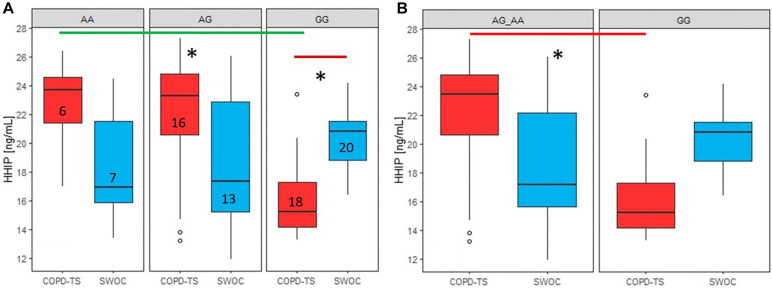
Comparison of serum HHIP levels between COPD-TS and SWOCgroups and recessive model by genotypes. AA: Common allele, AG: Heterozygous; GG: risk allele hoinozygous; COPD-TS: tobacco smoking patients; SWOC: Smokers without COPD: HHIP serum Protein levels are shown in ng/mL. Red lines show the comparison of Protein levels among COPD-TS vs. SWOC groups; Green lines represent the comparison between the genotypes in the COPD-TS group; ^∗^Statistically significant diferences.

For rs13147758, there were significant differences in HHIP serum protein levels between the homozygous genotypes (AA, p = 0.0086 and GG p = 0.0023); however, after adjusting for confounders, significance was only retained for the GG genotype (AA, *p* = 0.054 and GG *p* = 0.031). COPD patients with the AA genotype exhibited higher levels than controls. In contrast, those homozygous for the minor allele (G) displayed lower levels than smokers without COPD (Figure 3A). No statistically significant associations were identified for rs1828591, and these data can be found in Supplementary Tables S6A, S6B and Supplementary Figure S2.

In the COPD-TS group, for rs13147758, there were differences in correlations between serum HHIP concentrations among the three genotypes (adjusted *p* = 0.028). *Post hoc* analysis revealed that the differences were between the AA and GG genotypes (*p* = 0.0013) and the AG and GG genotypes (*p* = 0.0027) (Figure 3A). In contrast, there were no differences between the COPD-TS group and smokers without COPD (*p* > 0.05).

### Serum Protein Level Analysis Through Genetic Association Models

Recessive and dominant models were applied to compare protein levels between both groups of smokers for the three polymorphisms.

In the COPD-TS group, the recessive model for rs13147758 showed statistically significant differences (adjusted *p* = 0.016) (Figure 3B). Compared to patients who were heterozygous or homozygous for the common allele, COPD patients homozygous for the risk allele displayed reduced protein levels. The rest of the results by genetic models (dominant) for this SNP did not exhibit statistically significant values (*p* > 0.05), as can be seen in Supplementary Tables S6A,B and Supplementary Figures S3A,B.

### Correlation Between Protein Levels and Lung Function Parameters in Patients and Controls

**FIGURE 4:**
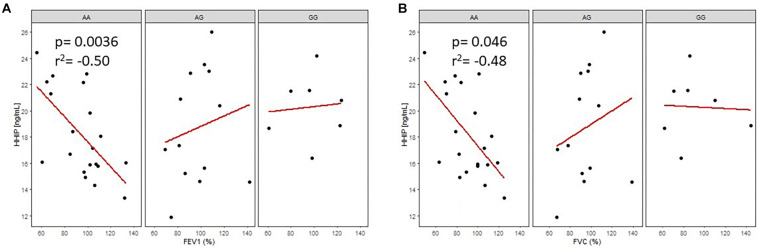
Correlation of HHIP protein levels in smokers without COPD (SWOC) with FEV_1_ (%) **A**, and FVC(%) **B**, according to the rsl3147758 genotypes. AA: Common allele, AG: Heterozygous; GG: risk allele homozygous.

As shown in Figures 4A,B, analysis of rs13147758 revealed negative correlations between protein levels and FEV_1_ and between protein levels and FVC in smokers without COPD. Those homozygous for the common allele (AA) demonstrated a negative correlation between protein levels and FEV_1_ (adjusted *p* = 0.036, *r*^2^ = −0.50) and between protein levels and FVC (adjusted *p* = 0.046, *r*^2^ = −0.48). The remaining correlations by genotype and in smokers with COPD were not statistically significant and are shown in Supplementary Tables S6A,B and Supplementary Figures S4A–D. In general, the correlations showed that lung function was improved in smokers without COPD with lower levels of serum protein.

For rs1828591, negative correlations between protein levels and FEV_1_ (adjusted *p* = 0.047, *r*^2^ = −0.50) and between protein levels and FVC (adjusted *p* = 0.048, *r*^2^ = −0.50) were observed in both cases in smokers without COPD with the AA genotype. In the group of smokers with COPD, the AA genotype was negatively correlated with serum protein levels and FEV_1_ (adjusted *p* = 0.035, *r*^2^ = −0.60). There were no statistically significant differences observed for the remaining correlations (*p* > 0.05, Supplementary Figures S6A–C, S7A,B).

### Sputum Supernatant HHIP Protein Levels in Smokers With and Without COPD

Next, a subgroup of 40 subjects (20 COPD-TS patients and 20 SWOC subjects) were selected to determine protein levels in the SS. Supplementary Table S5 shows the comparative demographic and clinical data used for this analysis.

**FIGURE 5:**
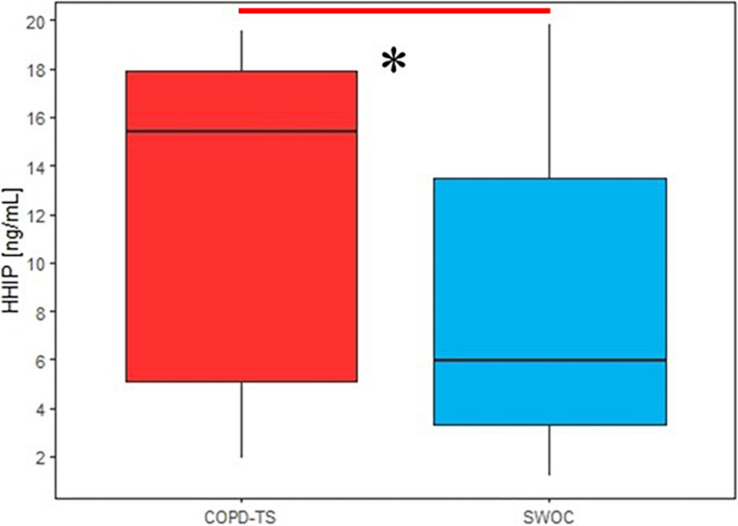
Comparison of sputum supernatant HHIP levels in smokers with COPD and without COPD secondary to tobacco smoking; HHIP sputum supernatant levels are expressed in ng/mL, ^∗^*p* < 0.05.

The first analysis was performed for all subjects in both groups, and the results demonstrated that HHIP protein levels in the SS were higher in the COPD-TS group then the SWOC group (*p* = 0.047, Figure 5).

### Comparison of Protein Levels Between the COPD-TS and SWOC Groups by Genotype and Genetic Models

Comparisons between the COPD-TS and SWOC groups based on rs13147758 genotypes revealed that smokers with COPD who were heterozygous (AG) exhibited higher protein levels in the SS then smokers without COPD (*p* = 0.19). There were no differences in protein levels in the SS among smokers with or without COPD of the three genotypes (*p* = 0.92 and *p* = 0.2, respectively). Furthermore, no differences in rs1828591 by genotype existed between the groups (*p* > 0.05). Of the two polymorphisms evaluated by genetic models, neither showed differences in recessive or dominant models (*p* > 0.05, Supplementary Tables S7A,B, S8A,B and Supplementary Figures S5A,B).

For rs13147758 and rs1828591, no significant differences were found in lung function parameters. Regarding the tobacco index, none of the genotypes of the different SNPs in either the patient or control groups showed statistically significant differences (Supplementary Tables S6B–S8B). Additionally, analysis of protein levels and genotype-specific GOLD grades were performed, and the results are shown in Supplementary Tables S6A–S8A.

## Discussion

In summary, the rs13147758 polymorphism is associated with COPD susceptibility, and codominant and recessive genetic models confirmed this finding. Furthermore, two haplotypes are associated with disease susceptibility, one with an increased risk (AG) and the other with a decreased disease risk (AA). Serum protein levels of COPD smokers harboring the rs13147758 GG genotype were lower than those in controls. In the intra-case analysis, COPD patients carrying the GG genotype exhibited reduced protein serum levels than those carrying the AA and AG genotypes, and this finding was reproduced with the recessive model, with the COPD-TS group (GG) exhibiting lower serum protein levels than the AA and AG genotypes. Finally, we identified negative correlations between protein levels and lung function parameters (FEV_1_ and FVC) among smokers without COPD possessing the AA genotype. Importantly, all statistically significant results after logistic regression analysis maintained the magnitude and direction of the association.

Chronic obstructive pulmonary disease is a multifactorial entity in which both environmental and genetic factors participate. Genetic markers have been correlated with a predisposition to developing COPD and pulmonary function alterations in different studies, primarily among smokers.

The demographic and clinical characteristics of the participants are critical for designing genetic association studies. In our research, we found statistically significant differences in age, sex, BMI, and tobacco index between the two groups. The association analysis was adjusted for these covariables using a logistical regression model. Of note, the differences in lung function parameters were expected because these parameters indicate whether individuals have COPD or not.

The BMI media of patients is lower (24.7) than the controls (27.4). Celli et al. (2004) reported that BMI is as good as FEV_1_ for predicting the risk of death from different respiratory causes, including COPD, and the negative effect of loss of body mass can be reversed in some patients with the right therapy (Schols et al., 1999).

Tobacco smoking is the leading risk factor for COPD. For both groups, the median age at which the subjects started smoking was ∼18 years (*p* > 0.05), and different reports indicate that beginning smoking in adolescence is considered a high-risk factor for the development of COPD. More than 90% of smokers begin consuming tobacco before the age of 20, with approximately17 million Mexicans between the ages of 12 and 65 years being smokers (Pérez-Rubio et al., 2015).

Molecular factors associated with a genetic predisposition to COPD have been widely studied (Pérez-Rubio et al., 2019). Several GWASs have identified a genetic loci of susceptibility, including *FAM13A*, *CHRNA5*/*3*, *IREB2*, *MMP3*/*MMP12*, *TGFB2*, and *HHIP*. The *HHIP* gene has been related to the FEV_1_/FVC ratio, FEV_1_, or both parameters of lung function, which are essential for diagnosis and COPD classification according to GOLD guidelines.

In the current analysis, SNPs that were commonly identified in reports and study populations were selected. Pillai et al. found that rs1828591 and rs13147758 in the *HHIP* gene were consistently identified across three different cohorts, but the combined p-values did not reach statistical significance at the GWAS level (1.47 × 10^–7^ and 1.67 × 10^–7^, respectively) (Pillai et al., 2009). In contrast, a GWAS by Wilk et al. identified some of the most important polymorphisms (rs13147758 and rs1828591) that reached statistical significance (*p* < 5 × 10^–8^) The SNP (rs13147758) identified with the association to the FEV_1_/FVC ratio was also statistically significant for FEV_1_ (*p* = 0.0001) and airflow obstruction (*p* = 6.18E-06) (Wilk et al., 2009).

In the current study, three genetic SNP variants (rs13147758, rs13118928, and rs1828591, all with MAF >5%) in the *HHIP* gene, were analyzed through a case–control study of smokers in the Mexican population. We found that the rs13147758 polymorphism was associated with susceptibility to COPD. Selected variants have been previously associated with lung function, including COPD, in different populations. For example, Xu et al. (2017) found that in Chinese populations (Han and Mongol), the homozygous genotype of the minor allele of rs13147758 has a protective role in the Han population, while the homozygous genotype of the minor allele rs1828591 has a protective role in both populations.

In the Rotterdam Study, Van Durme et al. (2010) showed that two polymorphisms (rs13118928 and rs1828591) are statistically associated with a decreased risk of developing COPD in Caucasian populations. Similarly, in a GWAS, Pillai et al. identified an association between these two polymorphisms and FEV_1_ in the BEOCOPD cohort and the British Birth Cohort; however, such associations were not found in the population in the ICGN (rs6817273, 2020). In the Framingham heart study, Wilk et al. (2009) reported that rs13147758 is statistically associated (p > 5 × 10^–8^) with the FEV_1_/FVC ratio and that homozygosity for the minor allele is significantly associated with FEV_1_ in smokers.

In our study, rs13147758 was evaluated in Mexican mestizo smokers, an association between genotypes and alleles, and the risk of COPD was found. Furthermore, individuals carrying the homozygous genotype for the minor allele (GG) exhibited an increased risk of suffering COPD (OR = 1.95). Analysis by association models shows that the codominant and recessive models maintain this association in both homozygous genotypes.

Haplotype analysis revealed that practically no linkage exists between the two polymorphisms analyzed, with the *r*^2^ values not reaching 0.80; however, we observed an *r*^2^ = 0.51 between rs1828591 and rs13147758. This could be due to the latest miscegenation process for Mexican mestizos. We also noted that the minor allele (G for both polymorphisms) exhibits differential frequencies among populations that were previously associated with COPD. For example, rs1828591 demonstrated the following minor allelic frequencies: CEU (Utah residents with Northern and Western European ancestry) = 0.434, CHB (Han Chinese in Bejing, China) = 0.320 and MXL (Mexican Ancestry in Los Angeles, California) = 0.305, rs13147758 occurred in CEU (Utah residents with Northern and Western European ancestry) = 0.429, CHB (Han Chinese in Bejing, China) = 0.316 and MXL (Mexican Ancestry in Los Angeles, California) = 0.328 (rs13147758, 2020a; rs1828591, 2020a). Caucasian populations have a higher frequency of the minor allele, and the risk of disease susceptibility is elevated in Caucasian populations (Pillai et al., 2009; Wilk et al., 2009). In our study, despite the frequency of the minor allele and the LD being smaller than in Caucasian studies, the same associations with susceptibility to COPD were observed.

Zhou and colleagues demonstrated that mRNA and protein levels are lower in the lungs of patients with COPD than in controls. A region (51 kb) near the *HHIP* gene acts as an enhancer, and there is a block of genetic variants (79 kb) that interact with the *HHIP* gene promoter through chromatin looping (Zhou et al., 2012; van der Plaat et al., 2017).

The Hhip protein, encoded by a gene with the same name, interacts with three members of the morphogen Hedgehog (Hh) family, which is highly conserved in mammals and is responsible for cellular differentiation during embryogenesis in vertebrates and invertebrates. In mammals, Hhip controls the neural tube patterns, limbs, somites, intestines, hair follicles, lungs, and even regulates the right-left symmetry of organisms (Murone et al., 1999; Bosanac et al., 2009). Some membrane proteins, cytoplasmic components, and transcriptional activators are essential for Hh signaling in the Hedgehog pathway. This signaling regulates many cellular processes, such as proteolysis, phosphorylation, and negative regulators (Chuang et al., 2003).

Two proteins are upregulated in response to hedgehog pathway activation: the hedgehog receptor protein patched (Ptch), which is vertebrate-specific, and Hedgehog interacting protein (*HHIP*). Both proteins form a negative feedback loop of hedgehog signaling necessary for the development of the lungs and other organs, since decreased protein levels are related to specific lung defects that result in neonatal lethality in a murine model (Wan et al., 2017) They are also involved in the response of pulmonary epithelial cells to smoking (Van Tuyl and Post, 2000; Chuang et al., 2003; Wang et al., 2013). This pathway plays a central role in the repair and regeneration of multiple tissues (Watkins et al., 2003; Rubin and de Sauvage, 2006; Wang et al., 2013).

Experimental studies have been performed in a murine *Hhip* haploinsufficient model to explore smoking as a risk factor for COPD. According to Wan et al., homozygous mice (*Hhip* −/−) die quickly after birth due to defects in their pulmonary morphogenesis, and while heterozygous mice (*Hhip* ±, haploinsufficient) are viable and exhibit normal pulmonary development, they do exhibit a ∼33% decrease in Hhip protein expression (Wan et al., 2017). However, no previous studies have described protein levels in the lung microenvironment in humans.

Several genetic association studies in COPD have found that, although SNPs in the *HHIP* gene are not associated with the disease *per se*, they are associated with some parameters of lung function, such as FEV_1_, FVC, and the FEV_1_/FVC ratio (Molfino, 2004). To evaluate the possible relationship of gene variants with more severe phenotypes, COPD patients were split into two clusters: G2 (more severe) and G1 (less severe). No differences were detected between these groups.

In the analysis of lung function and genotypes, we observed that when all individuals were grouped as smokers (regardless of COPD status), individuals who were homozygous for the minor allele (GG) of rs13147758, exhibited a decreased FEV_1_/FVC ratio compared to that of carriers of the common genotype (AA), supporting the hypothesis that smokers with the GG genotype exhibit worse lung function. Similarly, in the analysis of lung function by genetic models, the dominant model demonstrated that smokers carrying the GG genotype (risk allele) exhibited a lower FVC (*p* = 0.021) than those with the AA genotype. In the same genetic model, smokers with the GG genotype exhibited a decrease in the FEV_1_/FVC ratio (*p* = 0.009). These data are consistent with a report by Wilk et al., in which lung function was modestly associated with FEV_1_ in the Framingham samples and significantly associated with FEV_1_ in the Family Heart Study (Wilk et al., 2009).

Hedgehog interacting protein levels in the serum and induced SS were also studied. To date, there are no data in the literature on serum or SS HHIP protein levels in COPD. No differences in serum levels were observed in the case-control comparison.

Interestingly, for rs13147758, COPD patients harboring the GG genotype (risk genotype) demonstrated lower protein levels than those with the AA genotype. Similarly, in an intra-case analysis, patients with the GG genotype exhibited lower levels than homozygous and heterozygous individuals. Finally, applying the recessive model (GG vs. AA + AG), the same results were found. Taken together, these findings indicate that COPD patients with the GG genotype have reduced serum levels of the HHIP protein.

For rs1828591, one of the most highly associated polymorphisms in other populations (Lamontagne et al., 2013; Kerkhof et al., 2014; Bártholo et al., 2019), the case-control analysis showed that AA COPD patients have higher protein levels than control subjects. Serum protein levels analyzed in the dominant model confirmed an association with the AA genotype in the COPD-TS group (AA vs. AG + GG). In contrast, patients with the GG genotype displayed lower protein levels than control subjects, which is similar to our finding for rs13147758. However, this comparison was not statistically significant, likely due to the low MAF in the Mexican population (rs13118928, 2020a). Studying more subjects with the GG genotype may allow us to identify alterations in the protein levels in this genotype.

Furthermore, in the correlation analysis for both study groups, the AA genotype was negatively correlated with FEV_1_ and FEVC, indicating that low protein concentration results in improved lung function. We can also see when serum protein levels are high, the lung function of smokers (with or without COPD) harboring the AA genotype is worse. The possible explanation for the behavior of the correlations is the limited number of participants with the GG genotype (homozygous to minor allele); increasing the number of subjects with this genotype could help us to visualize better the behavior of the protein in the SWOC group.

The protein levels results were adjusted by sex, showing that this covariate does not interfere with the result, suggesting that HHIP protein levels are not dependent on the sex of the participants. Also, we identified less representation of women in our study groups, a phenomenon described among smokers in our previous studies.(Ambrocio-Ortiz et al., 2018)

Unlike serum levels, HHIP levels in the induced SS were statistically different between the COPD-TS and SWOC groups. Smokers with COPD demonstrated higher levels of the protein then SWOC, while in the serum, only intra-case differences were detected.

HHIP is a membrane protein that binds to Shh, Dhh, and Ihh to activate a signaling cascade, which is a crucial step for pulmonary embryonic development, and reduced HHIP gene expression has been observed in COPD pathogenesis (Zhou et al., 2013).

In contrast, compared to wild-type control, HHIP haploinsufficient mice exposed to cigarette smoke exhibit activation of lymphocytic pathways, increased activation of TCD8 cells (Lao et al., 2015), and more severe emphysema, suggesting that HHIP protects lung cells from environmental stressors, such as oxidative stress (Lao et al., 2016).

Although there are possible explanations for the role of the HHIP protein in emphysema physiopathology in murine models, the mechanism concerning COPD in humans remains to be elucidated.

Our results show that variants in the *HHIP* gene that are associated with genetic susceptibility are related to alterations in the serum and sputum protein levels of HHIP. However, whether protein levels in serum and sputum are the result of an active proteolytic process, perhaps as a defense against stressful agents like cigarette smoke, or the process of liberation products of alveolar destruction underlying emphysema remains unclear.

Analysis of different rs13147758 genotypes between patients and controls demonstrated that patients with the three genotypes exhibited higher protein levels in the SS than their respective controls. However a statistically significant difference was not shown. Importantly, comparisons in the induced SS were only made in twenty subjects from each group.

This study is not exempt from limitations. For example, the sample size was small compared to that of other studies, particularly the GWAS meta-analysis, and our participants were recruited from a single center. However, the power calculation for this sample size for genetic and protein analysis reached 77.9 and 78.4%, which we believe are good sample size powers for a validation study that evaluates novel correlations between serum and sputum protein levels and lung function parameters. Also, despite the current study being derived from a cohort where previously we have evaluated AIMs, and no statistical differences were found in the ancestry analysis, we were not able to assess genetic ancestry at an individual level, and this covariate was not included in the logistic regression model. Multicenter studies, including other mixed-race populations, would be desirable.

Finally, the three polymorphisms analyzed are the most replicated and strongly associated with COPD in Caucasian and Asian populations. However, to date, there is no precise information about the role of *HHIP* in COPD pathophysiology.

Our results suggest that homozygosity for the risk alleles for rs13147758 results in reduced serum protein levels and are thus genetic factors for the risk of developing COPD in Mexican mestizo smokers.

## Conclusion

Of the three evaluated polymorphisms in Mexican mestizo smokers, rs13147758 was associated with susceptibility to COPD and reduced lung function values among smokers. In addition, two haplotypes are associated with an increased COPD risk, which reinforces previous findings on the participation of genetic variants of *HHIP* in COPD and lung function.

The current study studied serum and sputum HHIP protein levels in smokers with and without COPD for the first time. Protein levels are associated with lung function, and variants in the *HHIP* gene regulate this gene’s participation in the risk of developing COPD.

## Data Availability Statement

The ClinVar accessions for the data in this article are SCV001245048, SCV001245049, SCV001245050.

## Ethics Statement

The studies involving human participants were reviewed and accepted by the Institutional Committees for Investigation, Ethics in Research, and Biosecurity of the Instituto Nacional de Enfermedades Respiratorias Ismael Cos o Villegas (INER) (approbation number: B11-19). The patients/participants provided their written informed consent to participate in this study.

## Author Contributions

RF-V: conceptualization. AO-M and EA-O: data curation. AO-M, GP-R, EA-O, KN-Q, and RF-V: formal analysis. RF-V: funding acquisition. AO-M, GP-R, KN-Q, RH-Z, SR-L, AH-P, LG-G, and RF-V: investigation. AO-M, EA-O, and KN-Q: methodology. RH-Z, EA-R, AR-V, and RF-V: project administration. EA-R, SR-L, AH-P, and LG-G: resources. AO-M and GP-R: software. GP-R, RH-Z, and RF-V: supervision. EA-R: visualization. AO-M, GP-R, AR-V and RF-V: writing – original draft. AO-M, AR-V, and RF-V: writing – review and editing. All authors contributed to the article and approved the submitted version.

## Conflict of Interest

The authors declare that the research was conducted in the absence of any commercial or financial relationships that could be construed as a potential conflict of interest.
