# Supplementary material for: The SNP rs13147758 in the HHIP Gene Is Associated With COPD Susceptibility, Serum, and Sputum Protein Levels in Smokers
Source: Front Genet. 2020 Sep 24;11:882. doi: 10.3389/fgene.2020.00882 (PMC7541950; doi:10.3389/fgene.2020.00882)
Supplement: Supplementary file 1 [file Data_Sheet_1.docx]

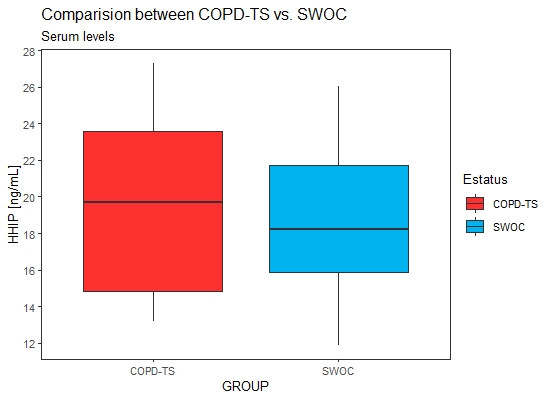


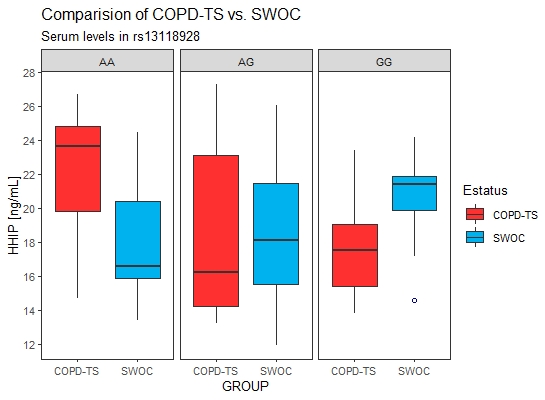
Supplementary figure S1. Comparison of serum HHIP levels in smokers with COPD and without COPD COPD-TS: COPD secondary to tobacco smoking; SWOC: Smokers without COPD; HHIP serum levels are expressed in ng/mL

Supplementary figure S2. Comparison of serum HHIP levels in smokers with COPD and without COPD; COPD-TS: COPD secondary to tobacco smoking; SWOC: Smokers without COPD by genotypes of the rs1828591; HHIP serum levels are expressed in ng/mL


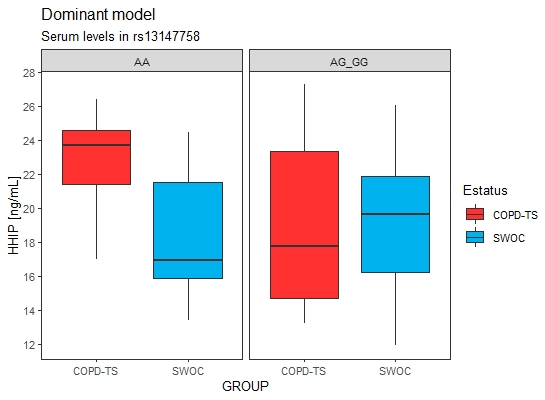

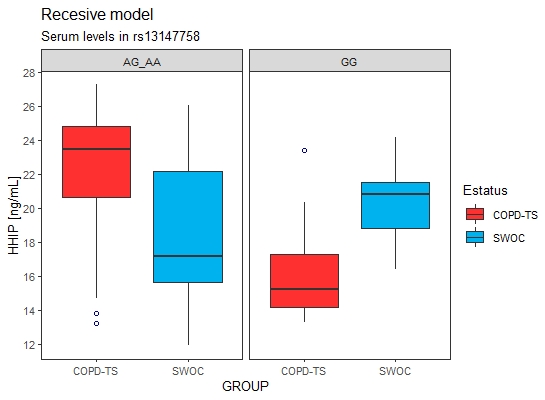


S3A

S3B

Supplementary Figures S3A, and S3B. Analysis of levels protein in the rs13147758 by the dominant and recessive genetic model. AA: Common allele homozygous; AG: Heterozygous genotype; GG: minor allele homozygous; COPD-TS: COPD tobacco smoking patients; SWOC: smokers without COPD. HHIP serum protein levels are shown in ng/mL.


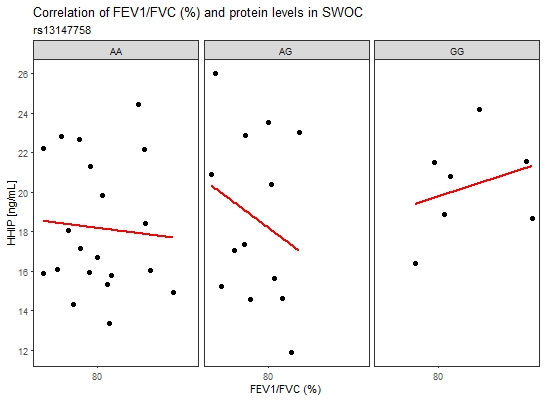

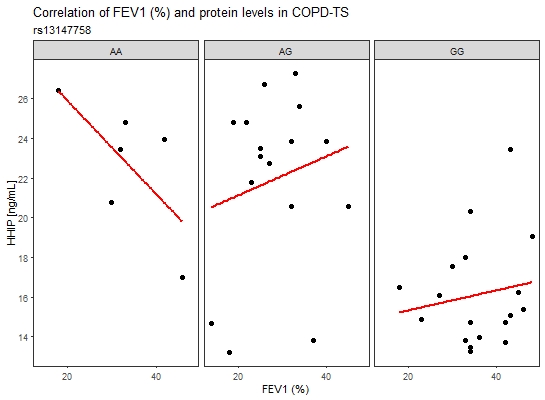

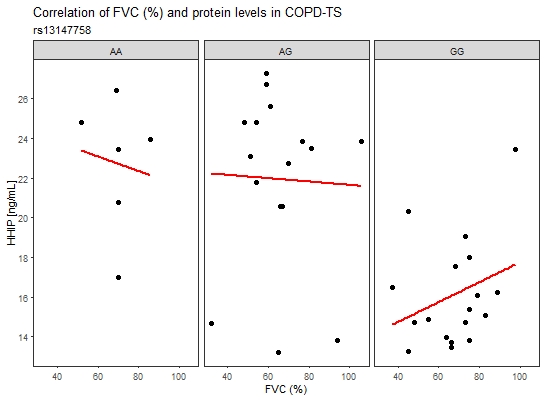

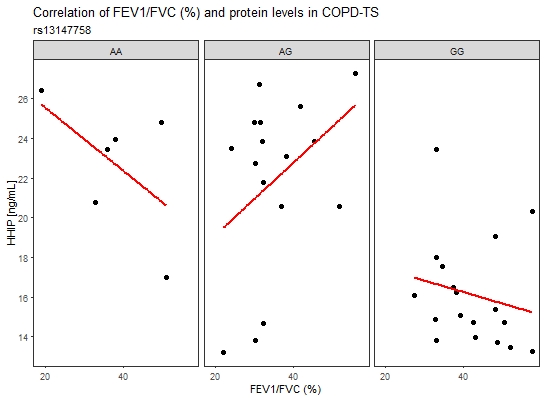


S4A

S4B

S4C

S4D

Supplementary figure S4A Correlation of protein levels in smokers without COPD (SWOC) with FEV1/FVC (%). (S4B) Correlation of protein levels in COPD tobacco smoking patients (COPD-TS) and FEV1 (%); (S4C) with FVC, and (S4D) FEV1/FVC (%) according to the rs13147758 genotypes. AA: Common allele homozygous; AG: Heterozygous genotype; GG: minor allele homozygous.


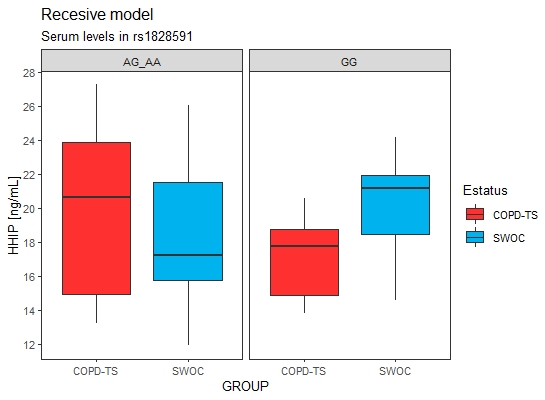

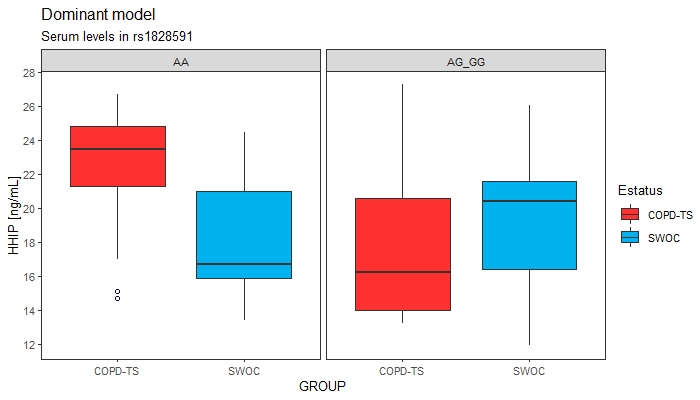


S5A

S5B

Supplementary Figures S5A, and S5B. Analysis of levels protein in the rs1828591 by the dominant and recessive genetic model. AA: Common allele homozygous; AG: Heterozygous genotype; GG: minor allele homozygous; COPD-TS: COPD tobacco smoking patients; SWOC: smokers without COPD. HHIP serum protein levels are shown in ng/mL.

Supplementary figure S6A Correlation of protein levels in smokers without COPD (SWOC) with FEV1/FVC (%). (S6B) Correlation of protein levels in COPD tobacco smoking patients (COPD-TS) and FVC (%); and (S6C) with FEV1/FVC according to the rs1828591 genotypes. AA: Common allele homozygous; AG: Heterozygous genotype; GG: minor allele homozygous.


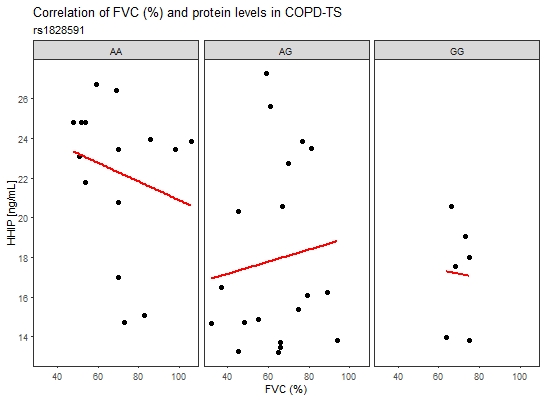

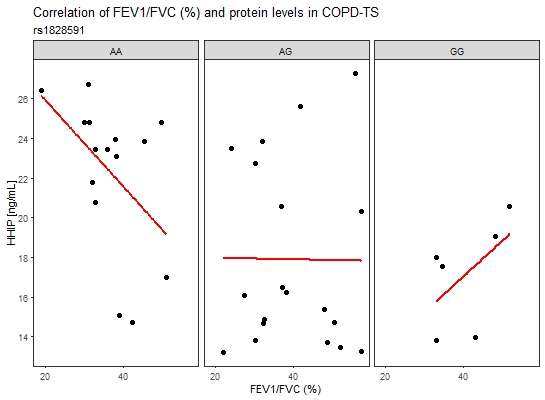

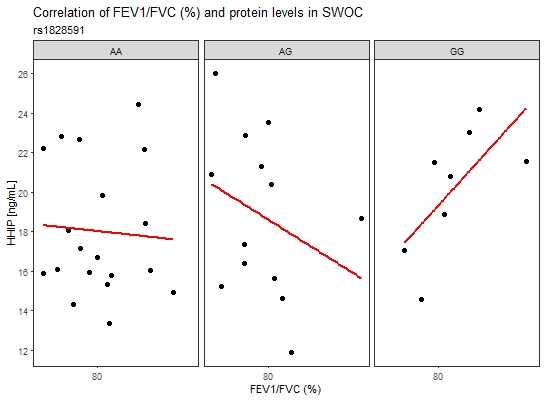


S6A

S6B

S6C


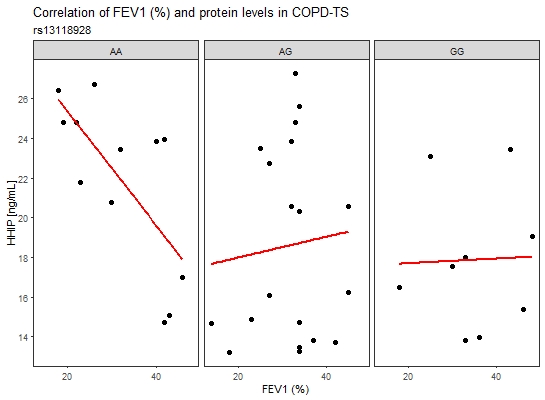

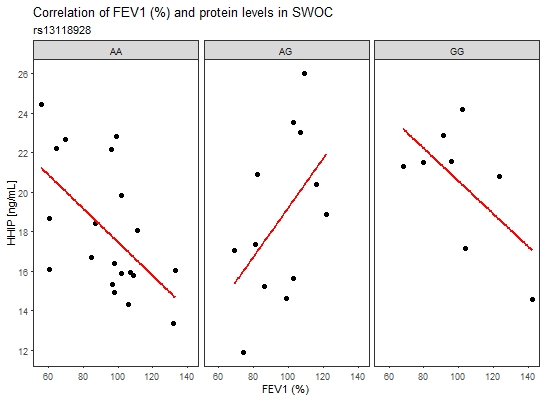

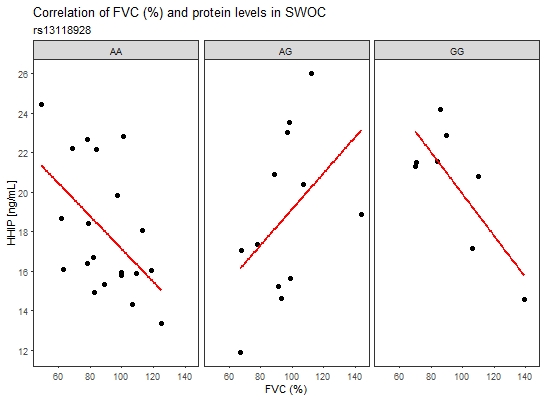


Figure 7. Correlation of Protein levels in smokers without COPD (SWOC) with FVC (%) 7A, FEV1(%) 7B, according to the rs13147758 genotypes, and correlation of protein levels in smokers with COPD (COPD-TS) with FEV1 (%). AA: Common allele, AG: Heterozygous; GG: risk allele homozygous.

S7A

S7B

S7C
