# Supplementary material for: The SNP rs13147758 in the HHIP Gene Is Associated With COPD Susceptibility, Serum, and Sputum Protein Levels in Smokers
Source: Front Genet. 2020 Sep 24;11:882. doi: 10.3389/fgene.2020.00882 (PMC7541950; doi:10.3389/fgene.2020.00882)
Supplement: Supplementary file 2 [file Data_Sheet_2.docx]

**Supplementary Table S1. General characteristics of SNPs included in the analysis**

| SNP | Position/  (Strand) | Ancestral  allele | Change | MAF | MXL | Gene  location |
| --- | --- | --- | --- | --- | --- | --- |
| rs13118928 | 144565237  (FWD) | A | A/G | G=0.30 | G=0.28 | Intron |
| rs1828591 | 144559628  (FWD) | A | A/G | G=0.41 | G=0.30 | Intron |
| rs13147758 | 144539078  (FWD) | A | A/G | G=0.29 | G=0.32 | Intron |

SNP Single Nucleotide Polymorphism, FWD Forward, MAF Minor Allele Frequency, MXL Mexican individuals from LA California.

**Supplementary Table S2. Analysis by genetics models of the rs1828591 of smokers with and without COPD.**

| Model/ Genotype | COPD-TS | | SWOC | | p-value | OR | 95% CI |
| --- | --- | --- | --- | --- | --- | --- | --- |
|  | n=222 | % | n=333 | % |  |  |  |
| Codominant |  |  |  |  |  |  |  |
| AA | 87 | 39.19 | 143 | 42.94 | 0.67* | 1 (ref.) |  |
| AG | 100 | 45.05 | 141 | 42.34 |  | 1.16 | 0.80 – 1.68 |
| GG | 35 | 15.76 | 49 | 14.72 |  | 1.17 | 0.70 – 1.95 |
| Recessive |  |  |  |  |  |  |  |
| GG  AA + AG | 35 | 15.76 | 49 | 14.71 | 0.80* | 1.08 | 0.67 – 1.73 |
|  | 187 | 84.24 | 284 | 85.29 |  | 0.92 | 0.57 – 1.47 |

COPD-TS: COPD tobacco smoking patients; SWOC: Smokers without COPD; SNP: Single Nucleotide Polymorphism; OR: odds ratio; 95% CI: 95% confidence interval. Statistically significant p-value <0.05; *p-value adjusted by age, sex, and tobacco index in the recessive model.

**Supplementary Table S3. Comparison among groups of patients with COPD and GOLD stages, G2 (GOLD III + IV) vs. G1 (GOLD I +II)**

| SNP | Genotype/  Allele | G2 | |  | G1 | | p-value | OR | 95% CI |
| --- | --- | --- | --- | --- | --- | --- | --- | --- | --- |
|  |  | **n=84** | **%** |  | **n=138** | **%** |  |  |  |
| rs13147758 | AA | 24 | 28.60 |  | 47 | 34.05 | 0.45 | 0.77 | 0.42 – 1.39 |
|  | AG | 39 | 46.40 |  | 54 | 39.13 | 0.32 | 1.34 | 0.77 - 2.33 |
|  | GG | 21 | 25.00 |  | 37 | 28.82 | 0.87 | 0.90 | 0.48 – 1.69 |
|  | A | 87 | 51.80 |  | 148 | 53.63 | 0.76 | 0.91 | 0.58 – 1.45 |
|  | G | 81 | 48.20 |  | 128 | 46.37 |  | 1.07 | 0.73 - 1.58 |
| rs1828591 | AA | 36 | 42.86 |  | 50 | 36.23 | 0.39 | 1.32 | 0.75 – 2.29 |
|  | AG | 35 | 41.67 |  | 65 | 47.10 | 0.48 | 0.80 | 0.46 – 1.38 |
|  | GG | 13 | 15.47 |  | 23 | 16.67 | 0.85 | 0.91 | 0.43 – 1.92 |
|  | A | 107 | 63.70 |  | 165 | 59.78 | 0.42 | 1.18 | 0.79 – 1.75 |
|  | G | 61 | 36.30 |  | 111 | 40.22 |  | 0.84 | 0.57 – 1.25 |

SNP: Single Nucleotide Polymorphism; G1 (GOLD I + II); G2 (GOLD III + IV); p statistically significant (<0.05); OR odds ratio; 95% CI confidence interval of 95%.

**Supplementary Table S4. Clinical and demographic characteristics of smokers with and without COPD of the subgroup for protein levels in serum.**

|  | COPD-TS n = 40 | SWOC n = 40 | p-value |
| --- | --- | --- | --- |
| Age | 67 (60 - 85) | 67 (49 - 78) | 0.29 |
| Male (%) | 36 (90) | 20 (50) | <0.01 |
| Female (%) | 4 (10) | 20 (50) | <0.01 |
| Tobacco Index | 33.6 (10.5 - 106) | 18.8 (10 - 120) | <0.01 |
| FEV_1_ | 33 (14 - 48) | 98.5 (56 - 142) | <0.01 |
| FVC | 67.5 (32 - 106) | 90.5 (49 - 144) | <0.01 |
| FEV_1_/FVC | 37.1 (19 - 57.5) | 80.05 (70.3 - 96) | <0.01 |
| GOLD III (%) | 25 (62.5) | NA | - |
| GOLD IV (%) | 15 (37.5) | NA | - |

COPD-TS: COPD tobacco smoking patients; SWOC: smokers without COPD; FEV_1_: Forced Expiratory Volume in the first second; FVC: Forced Vital Capacity; Statistically significant p-value <0.05; NA: Not apply. Showing the mean and min-max values

**Supplementary Table S5. Clinical and demographic characteristics of smokers with and without COPD of the subgroup for protein levels in sputum supernatant.**

|  | COPD-TS n = 20 | SWOC n = 20 | p-value |
| --- | --- | --- | --- |
| Age | 70 (45 – 88) | 68 (45 – 85) | 0.29 |
| Male (%) | 13 (65) | 5 (25) | <0.01 |
| Female (%) | 7 (35) | 15 (75) | <0.01 |
| Tobacco Index | 33.75 (19 – 82) | 30.25 (17 – 56) | <0.01 |
| FEV_1_ | 62 (45 – 89) | 95 (59 – 115) | <0.01 |
| FVC | 80 (62 – 111) | 94 (62 – 118) | <0.01 |
| FEV_1_/FVC | 54 (35 – 68) | 102 (76 – 112) | <0.01 |
| GOLD I (%) | 4 (20) | NA | NA |
| GOLD II (%) | 13 (65) | NA | NA |
| GOLD III (%) | 3 (15) | NA | NA |

COPD-TS: COPD tobacco smoking patients; SWOC: smokers without COPD; FEV_1_: Forced Expiratory Volume in the first second; FVC: Forced Vital Capacity; Statistically significant p-value <0.05; NA: Not apply. Showing the mean and min-max values

**Supplementary Table S6A. Analysis of serum HHIP protein levels among smokers with and without COPD, according to rs13147758 genotypes.**

| rs13147758 | COPD-TS  n = 40 | SWOC  n = 40 | p-value |
| --- | --- | --- | --- |
| Protein levels [ng/mL] | 19.68 (13.22 – 27.27) | 18.24 (11.91 – 26.02) | 0.582 |
| Genotypes |  |  |  |
| AA | 23.70 (17 – 26.39) | 16.93 (13.39 – 24.44) | 0.0086 |
| AG | 23.30 (13.22 – 27.27) | 17.36 (11.91 – 26.02) | 0.0616 |
| GG | 15.24 (13.28 – 23.42) | 20.80 (16.41 – 24.18) | 0.0023 |
| COPD-TS | AA vs. AG vs. GG | | 0.0005 |
|  | AA vs. AG | | 1 |
|  | AA vs. GG | | 0.0013 |
|  | AG vs. GG | | 0.01027 |
| SWOC | AA vs. AG vs. GG | | 0.3516 |
|  | AA vs. AG | | 1 |
|  | AA vs. GG | | 0.39 |
|  | AG vs. GG | | 1 |
| Genetic models | | | |
| Recessive, COPD-TS | GG vs. AA+AG | | 5.30E-05 |
| Dominant, COPD-TS | AA vs. AG+GG | | 0.0493 |
| Recessive, SWOC | GG vs. AA+AG | | 0.1687 |
| Dominant, SWOC | AA vs. AG+GG | | 0.3547 |
| GOLD stage vs. SWOC | | | |
| AA (p = 0.027) | SWOC | G-III | 0.24 |
|  | SWOC | G-IV | 0.052 |
|  | G-IV | G-III | 1 |
| AG (p = 0.16) | SWOC | G-III | 0.34 |
|  | SWOC | G-IV | 0.43 |
|  | G-IV | G-III | 1 |
| GG (p = 0.012) | SWOC | G-III | 0.017 |
|  | SWOC | G-IV | 0.073 |
|  | G-IV | G-III | 1 |
| GOLD III and IV by genotypes | | | |
| GOLD III | AA vs. AG vs. GG | | 0.0053 |
|  | AA vs. AG | | 1 |
|  | AA vs. GG | | 0.035 |
|  | AG vs. GG | | 0.017 |
| GOLD IV | AA vs. AG vs. GG | | 0.1194 |
|  | AA vs. AG | | 1 |
|  | AA vs. GG | | 0.4 |
|  | AG vs. GG | | 0.44 |

AA: Common allele homozygous; AG: Heterozygous; GG: risk allele homozygous;COPD-TS: COPD tobacco smoking patients; SWOC: smokers without COPD; FEV_1_: Forced Expiratory Volume in the first second; FVC: Forced Vital Capacity; Statistically significant p-value <0.05; Showing the mean and min-max values

**Supplementary Table S6B. Lung function correlations with serum HHIP concentrations by genotypes in the study groups for rs13147758**

| rs13147758 | Genotype | p-value | r^2^ |
| --- | --- | --- | --- |
| SWOC |  |  |  |
| FEV_1_ | AA | 0.0033 | -0.6226 |
|  | AG | 0.5581 | 0.1791 |
|  | GG | 0.8492 | 0.0891 |
| FVC | AA | 0.0070 | -0.5825 |
|  | AG | 0.4635 | 0.2232 |
|  | GG | 0.9202 | -0.0470 |
| FEV_1_/FVC | AA | 0.7622 | -0.0722 |
|  | AG | 0.4070 | -0.2515 |
|  | GG | 0.5172 | 0.2973 |
| COPD-TS |  |  |  |
| FEV_1_ | AA | 0.1323 | -0.6861 |
|  | AG | 0.4844 | 0.1885 |
|  | GG | 0.5422 | 0.1538 |
| FVC | AA | 0.8245 | -0.1175 |
|  | AG | 0.8999 | -0.0342 |
|  | GG | 0.2227 | 0.3023 |
| FEV_1_/FVC | AA | 0.2481 | -0.5597 |
|  | AG | 0.1405 | 0.3853 |
|  | GG | 0.4343 | -0.1966 |
| SWOC |  |  |  |
| Tobacco index | AA | 0.7193 | 0.0857 |
|  | AG | 0.4999 | -0.0258 |
|  | GG | 0.6243 | -0.2271 |
| COPD-TS |  |  |  |
| Tobacco index | AA | 0.8309 | 0.1132 |
|  | AG | 0.2656 | -0.2960 |
|  | GG | 0.3859 | -0.2175 |

COPD-TS: COPD tobacco smoking patients; SWOC: smokers without COPD; FEV_1_: Forced Expiratory Volume in the first second; FVC: Forced Vital Capacity; r^2^: correlation Statistically significant p-value <0.05.

**Supplementary Table S7A Analysis of sputum supernatant HHIP protein levels among smokers with and without COPD according to rs13147758 genotypes.**

| rs13147758 | COPD-TS  n = 20 | SWOC  n = 20 | p-value |
| --- | --- | --- | --- |
| Protein levels [ng/mL] | 15.37 (1.93 – 19.59) | 5.93 (1.23 – 19.8) | 0.036 |
| Genotypes |  |  |  |
| AA | 15.37 (1.93 – 19.59) | 5.91 (2.10 – 19.80) | 0.56 |
| AG | 15.58 (3.30 – 18.96) | 3.86 (1.23 – 12.13) | 0.026 |
| GG | 14.76 (3.13 – 17.90) | 10.39 (4.73 – 17.04) | 0.53 |
| COPD-TS | AA vs. AG vs. GG | | 0.92 |
|  | AA vs. AG | | 1 |
|  | AA vs. GG | | 1 |
|  | AG vs. GG | | 1 |
| SWOC | AA vs. AG vs. GG | | 0.2 |
|  | AA vs. AG | | 1 |
|  | AA vs. GG | | 0.2 |
|  | AG vs. GG | | 1 |
| Genetic models | | | |
| Recessive, COPD-TS | GG vs. AA+AG | | 0.72 |
| Dominant, COPD-TS | AA vs. AG+GG | | 0.93 |
| Recessive, SWOC | GG vs. AA+AG | | 0.17 |
| Dominant, SWOC | AA vs. AG+GG | | 0.81 |
| GOLD stage vs. SWOC | | | |
| AA (p = 0.36) | SWOC | G-II | 1 |
|  | SWOC | G-III | 1 |
|  | G-II | G-III | 0.75 |
| AG (p = 0.13) | SWOC | G-I | 1 |
|  | SWOC | G-II | 0.25 |
|  | SWOC | G-III | 1 |
|  | G-I | G-II | 1 |
|  | G-I | G-III | 1 |
|  | G-II | G-III | 1 |
| GG (p = 0.20) | SWOC | G-I | 1 |
|  | SWOC | G-II | 1 |
|  | SWOC | G-III | 1 |
|  | G-I | G-II | 1 |
|  | G-I | G-III | 1 |
|  | G-II | G-III | 1 |

AA: Common allele homozygous; AG: Heterozygous; GG: risk allele homozygous;COPD-TS: COPD tobacco smoking patients; SWOC: smokers without COPD; FEV_1_: Forced Expiratory Volume in the first second; FVC: Forced Vital Capacity; Statistically significant p-value <0.05; Showing the mean and min-max values

**Supplementary Table S7B. Lung function correlations with sputum supernatant HHIP concentrations by genotypes in the study groups for rs13147758**

| rs13147758 | Genotype | p-value | r^2^ |
| --- | --- | --- | --- |
| SWOC |  |  |  |
| FEV_1_ | AA | 0.32 | 0.43 |
|  | AG | 0.11 | -0.65 |
|  | GG | 0.76 | 0.16 |
| FVC | AA | 0.67 | 0.19 |
|  | AG | 0.94 | -0.03 |
|  | GG | 0.71 | 0.19 |
| FEV_1_/FVC | AA | 0.2 | 0.54 |
|  | AG | 0.45 | -0.33 |
|  | GG | 0.29 | 0.51 |
| COPD-TS |  |  |  |
| FEV_1_ | AA | 0.15 | -0.55 |
|  | AG | 0.72 | -0.16 |
|  | GG | 0.16 | -0.72 |
| FVC | AA | 0.22 | -0.48 |
|  | AG | 0.94 | 0.03 |
|  | GG | 0.61 | -0.03 |
| FEV_1_/FVC | AA | 0.64 | 0.19 |
|  | AG | 0.75 | -0.14 |
|  | GG | 0.17 | -0.72 |
| SWOC |  |  |  |
| Tobacco index | AA | 0.84 | 0.09 |
|  | AG | 0.14 | -0.61 |
|  | GG | 0.15 | 0.65 |
| COPD-TS |  |  |  |
| Tobacco index | AA | 0.079 | -0.65 |
|  | AG | 0.29 | 0.46 |
|  | GG | 0.60 | 0.31 |

COPD-TS: COPD tobacco smoking patients; SWOC: smokers without COPD; FEV_1_: Forced Expiratory Volume in the first second; FVC: Forced Vital Capacity; r^2^: correlation Statistically significant p-value <0.05.

**Supplementary Table S8A. Analysis of sputum supernatant HHIP protein levels among smokers with and without COPD, according to rs1828591 genotypes.**

| rs1828591 | COPD-TS  n = 20 | SWOC  n = 20 | p-value |
| --- | --- | --- | --- |
| Protein levels [ng/mL] | 15.37 (1.93 – 19.59) | 5.93 (1.23 – 19.8) | 0.036 |
| Genotypes |  |  |  |
| AA | 14.76 (3.04 – 19.59) | 15.24 (2.13 – 19.80) | 1 |
| AG | 15.35 (3.13 – 19.09) | 5.93 (1.77 – 15.58) | 0.49 |
| GG | 15.58 (1.93 – 18.28) | 4.73 (1.23 – 8.51) | 0.3 |
| COPD-TS | AA vs. AG vs. GG | | 0.94 |
|  | AA vs. AG | | 1 |
|  | AA vs. GG | | 1 |
|  | AG vs. GG | | 1 |
| SWOC | AA vs. AG vs. GG | | 0.12 |
|  | AA vs. AG | | 0.62 |
|  | AA vs. GG | | 0.29 |
|  | AG vs. GG | | 0.76 |
| Genetic models | | | |
| Recessive, COPD-TS | GG vs. AA+AG | | 0.82 |
| Dominant, COPD-TS | AA vs. AG+GG | | 0.93 |
| Recessive, SWOC | GG vs. AA+AG | | 0.11 |
| Dominant, SWOC | AA vs. AG+GG | | 0.09 |
| GOLD stage vs. SWOC | | | |
| AA (p = 0.11) | SWOC | G-II | 1 |
|  | G-II | G-III | 1 |
| AG (p = 0.39) | SWOC | G-I | 1 |
|  | SWOC | G-II | 0.025 |
|  | G-I | G-II | 1 |
| GG (p = 0.26) | SWOC | G-II | 1 |
|  | SWOC | G-III | 1 |
|  | G-II | G-III | 1 |

AA: Common allele homozygous; AG: Heterozygous; GG: risk allele homozygous;COPD-TS: COPD tobacco smoking patients; SWOC: smokers without COPD; FEV_1_: Forced Expiratory Volume in the first second; FVC: Forced Vital Capacity; Statistically significant p-value <0.05; Showing the mean and min-max values

**Supplementary Table S8B. Lung function correlations with sputum supernatant HHIP concentrations by genotypes in the study groups for rs1828591**

| rs1828591 | Genotype | p-value | r^2^ |
| --- | --- | --- | --- |
| SWOC |  |  |  |
| FEV_1_ | AA | 0.91 | -0.06 |
|  | AG | 0.73 | 0.12 |
|  | GG | 0.81 | 0.14 |
| FVC | AA | 0.73 | -0.21 |
|  | AG | 0.81 | -0.08 |
|  | GG | 0.54 | -0.36 |
| FEV_1_/FVC | AA | 0.7 | 0.23 |
|  | AG | 0.42 | 0.28 |
|  | GG | 0.53 | 0.37 |
| COPD-TS |  |  |  |
| FEV_1_ | AA | 0.07 | -0.83 |
|  | AG | 0.18 | -0.45 |
|  | GG | 0.67 | -0.26 |
| FVC | AA | 0.13 | -0.75 |
|  | AG | 0.95 | -0.02 |
|  | GG | 0.59 | -0.32 |
| FEV_1_/FVC | AA | 0.40 | 0.48 |
|  | AG | 0.53 | -0.22 |
|  | GG | 0.64 | -0.28 |
| SWOC |  |  |  |
| Tobacco index | AA | 0.48 | 0.41 |
|  | AG | 0.64 | 0.16 |
|  | GG | 0.31 | -0.56 |
| COPD-TS |  |  |  |
| Tobacco index | AA | 0.29 | -0.58 |
|  | AG | 0.76 | -0.10 |
|  | GG | 0.58 | 0.33 |

COPD-TS: COPD tobacco smoking patients; SWOC: smokers without COPD; FEV_1_: Forced Expiratory Volume in the first second; FVC: Forced Vital Capacity; r^2^: correlation Statistically significant p-value <0.05.
